# Supplementary material for: Leveraging gene correlations in single cell transcriptomic data
Source: BMC Bioinformatics. 2024 Sep 18;25:305. doi: 10.1186/s12859-024-05926-z (PMC11411778; doi:10.1186/s12859-024-05926-z)
Supplement: Supplementary file 3 — Additional file 3: Figure S1. Significance of uncorrected Pearson correlation coefficients, as calculated by BigSur versus the Fisher formula, binned by gene expression. scRNAseq data were as described in Fig. 3. Data points representing pairs of genes were divided into 21 bins based on the mean expression levels of each gene, and the results for each bin were plotted as described in Fig. 3C. The abscissa shows PCCwhile the ordinate gives the negative log10 of p values determined by BigSur, i.e., larger values mean greater statistical significance. Orange and gray shading indicate gene pairs judged significant by BigSur. Blue and orange show gene pairs that would have been judged statistically significant by applying the Fisher formula to the PCC, using the same p-value threshold as used by BigSur. The blue region contains gene pairs judged significant by the Fisher formula only, while the unshaded region shows gene pairs not significant by either method. Numbers in the lower right corner of each panel are the total numbers of possible correlations, statistically significant correlations according to the Fisher formula, and statistically significant correlations according to BigSur. [file 12859_2024_5926_MOESM3_ESM.pdf]

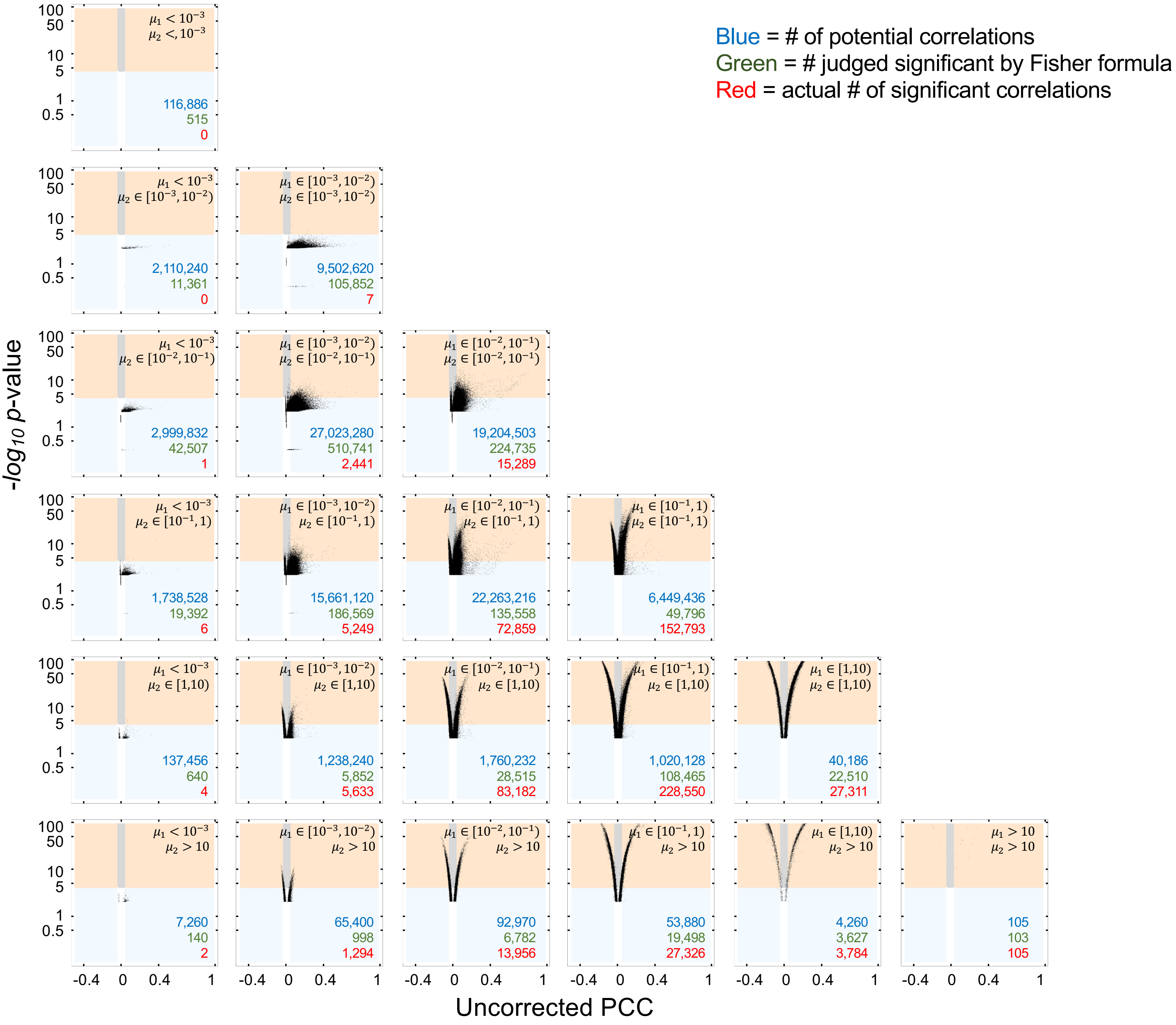
Blue = # of potential correlations
Green = # judged significant by Fisher formula
Red = actual # of significant correlations
-log10 p-value
Uncorrected PCC
100
50
10
5
1
0.5
-0.4
0
0.4
1
$\mu_1 < 10^{-3}$ $\mu_2 <, 10^{-3}$
116,886
515
0
$\mu_1 < 10^{-3}$ $\mu_2 \in [10^{-3}, 10^{-2})$
2,110,240
11,361
0
$\mu_1 \in [10^{-3}, 10^{-2})$ $\mu_2 \in [10^{-3}, 10^{-2})$
9,502,620
105,852
7
$\mu_1 < 10^{-3}$ $\mu_2 \in [10^{-2}, 10^{-1})$
2,999,832
42,507
1
$\mu_1 \in [10^{-3}, 10^{-2})$ $\mu_2 \in [10^{-2}, 10^{-1})$
27,023,280
510,741
2,441
$\mu_1 \in [10^{-2}, 10^{-1})$ $\mu_2 \in [10^{-2}, 10^{-1})$
19,204,503
224,735
15,289
$\mu_1 < 10^{-3}$ $\mu_2 \in [10^{-1}, 1)$
1,738,528
19,392
6
$\mu_1 \in [10^{-3}, 10^{-2})$ $\mu_2 \in [10^{-1}, 1)$
15,661,120
186,569
5,249
$\mu_1 \in [10^{-2}, 10^{-1})$ $\mu_2 \in [10^{-1}, 1)$
22,263,216
135,558
72,859
$\mu_1 \in [10^{-1}, 1)$ $\mu_2 \in [10^{-1}, 1)$
6,449,436
49,796
152,793
$\mu_1 < 10^{-3}$ $\mu_2 \in [1,10)$
137,456
640
4
$\mu_1 \in [10^{-3}, 10^{-2})$ $\mu_2 \in [1,10)$
1,238,240
5,852
5,633
$\mu_1 \in [10^{-2}, 10^{-1})$ $\mu_2 \in [1,10)$
1,760,232
28,515
83,182
$\mu_1 \in [10^{-1}, 1)$ $\mu_2 \in [1,10)$
1,020,128
108,465
228,550
$\mu_1 \in [1,10)$ $\mu_2 \in [1,10)$
40,186
22,510
27,311
$\mu_1 < 10^{-3}$ $\mu_2 > 10$
7,260
140
2
$\mu_1 \in [10^{-3}, 10^{-2})$ $\mu_2 > 10$
65,400
998
1,294
$\mu_1 \in [10^{-2}, 10^{-1})$ $\mu_2 > 10$
92,970
6,782
13,956
$\mu_1 \in [10^{-1}, 1)$ $\mu_2 > 10$
53,880
19,498
27,326
$\mu_1 \in [1,10)$ $\mu_2 > 10$
4,260
3,627
3,784
$\mu_1 > 10$ $\mu_2 > 10$
105
103
105

Figure S1. Significance of uncorrected Pearson correlation coefficients (PCC), as calculated by BigSur versus the Fisher formula, binned by gene expression. scRNAseq data were as described in Figure 3. Data points representing pairs of genes were divided into 21 bins based on the mean expression levels of each gene, and the results for each bin were plotted as described in Fig. 3C. The abscissa shows PCC (calculated from default-normalized data) while the ordinate gives the negative log_{10} of *p*-values determined by BigSur, i.e., larger values mean greater statistical significance. Orange and gray shading indicate gene pairs judged significant by BigSur (FDR<0.02). Blue and orange show gene pairs that would have been judged statistically significant by applying the Fisher formula to the PCC, using the same *p*-value threshold as used by BigSur. The blue region contains gene pairs judged significant by the Fisher formula only, while the unshaded region shows gene pairs not significant by either method. Numbers in the lower right corner of each panel are the total numbers of possible correlations (blue), statistically significant correlations according to the Fisher formula (green), and statistically significant correlations according to BigSur (red).
