# Supplementary material for: Leveraging gene correlations in single cell transcriptomic data
Source: BMC Bioinformatics. 2024 Sep 18;25:305. doi: 10.1186/s12859-024-05926-z (PMC11411778; doi:10.1186/s12859-024-05926-z)
Supplement: Supplementary file 4 — Additional file 4: Figure S2. Significance of modified corrected Pearson correlation coefficients,, as calculated by BigSur versus the Fisher formula, binned by gene expression. scRNAseq data were as described in Fig. 3. Data points representing pairs of genes were divided into 21 bins based on the mean expression levels of each gene, and the results for each bin were plotted as in Fig. S1. The inset compares the PCC′-p value relationship determined by BigSurwith that predicted by the Fisher formula, showing that, for highly expressed genes, the two methods agree well. [file 12859_2024_5926_MOESM4_ESM.pdf]

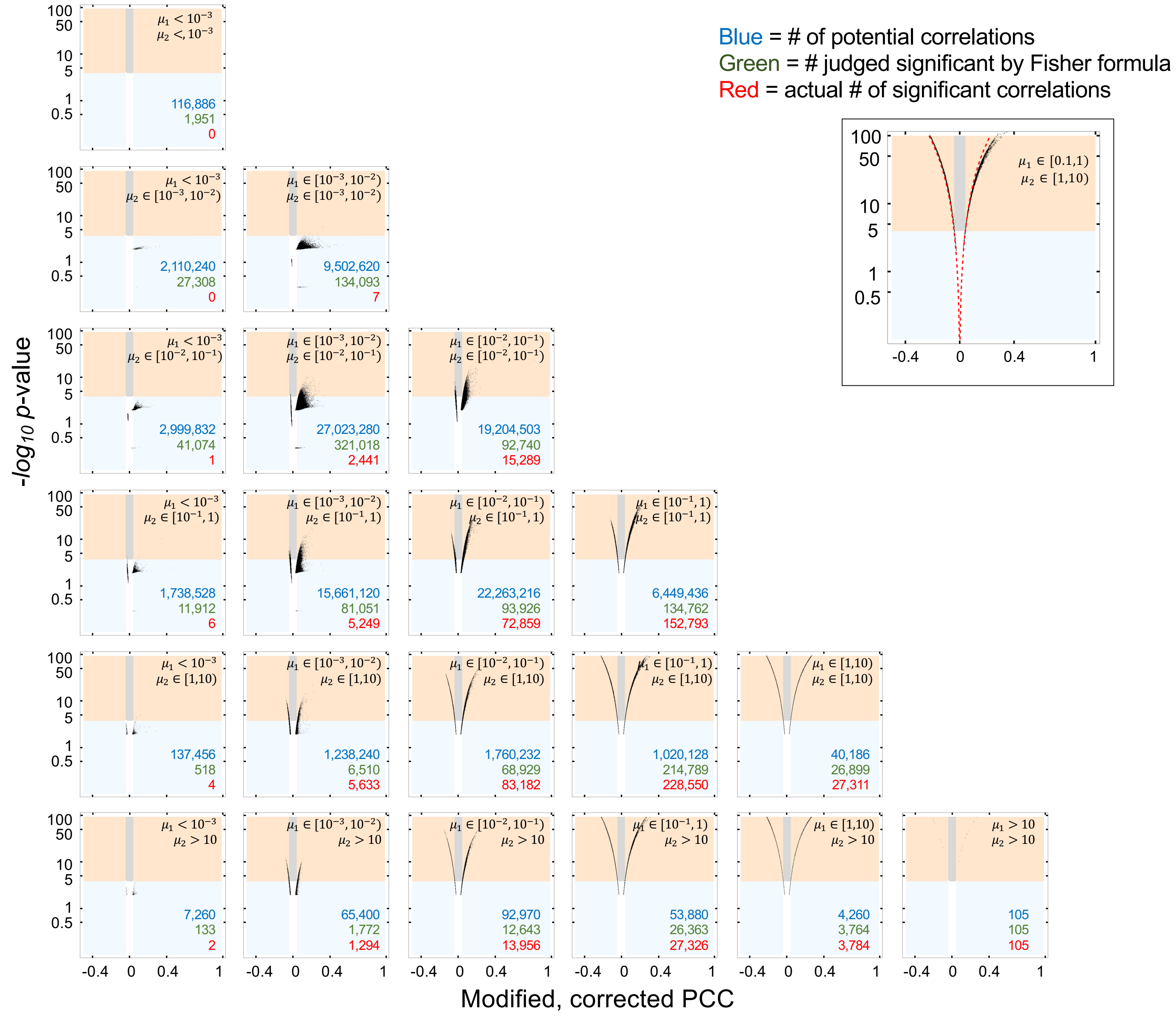

Blue = # of potential correlations
Green = # judged significant by Fisher formula
Red = actual # of significant correlations
-log10 p-value
Modified, corrected PCC
μ1 < 10^-3 μ2 <, 10^-3
116,886
1,951
0
μ1 < 10^-3 μ2 ∈ [10^-3, 10^-2)
2,110,240
27,308
0
μ1 ∈ [10^-3, 10^-2) μ2 ∈ [10^-3, 10^-2)
9,502,620
134,093
7
μ1 < 10^-3 μ2 ∈ [10^-2, 10^-1)
2,999,832
41,074
1
μ1 ∈ [10^-3, 10^-2) μ2 ∈ [10^-2, 10^-1)
27,023,280
321,018
2,441
μ1 ∈ [10^-2, 10^-1) μ2 ∈ [10^-2, 10^-1)
19,204,503
92,740
15,289
μ1 < 10^-3 μ2 ∈ [10^-1, 1)
1,738,528
11,912
6
μ1 ∈ [10^-3, 10^-2) μ2 ∈ [10^-1, 1)
15,661,120
81,051
5,249
μ1 ∈ [10^-2, 10^-1) μ2 ∈ [10^-1, 1)
22,263,216
93,926
72,859
μ1 ∈ [10^-1, 1) μ2 ∈ [10^-1, 1)
6,449,436
134,762
152,793
μ1 < 10^-3 μ2 ∈ [1,10)
137,456
518
4
μ1 ∈ [10^-3, 10^-2) μ2 ∈ [1,10)
1,238,240
6,510
5,633
μ1 ∈ [10^-2, 10^-1) μ2 ∈ [1,10)
1,760,232
68,929
83,182
μ1 ∈ [10^-1, 1) μ2 ∈ [1,10)
1,020,128
214,789
228,550
μ1 ∈ [1,10) μ2 ∈ [1,10)
40,186
26,899
27,311
μ1 < 10^-3 μ2 > 10
7,260
133
2
μ1 ∈ [10^-3, 10^-2) μ2 > 10
65,400
1,772
1,294
μ1 ∈ [10^-2, 10^-1) μ2 > 10
92,970
12,643
13,956
μ1 ∈ [10^-1, 1) μ2 > 10
53,880
26,363
27,326
μ1 ∈ [1,10) μ2 > 10
4,260
3,764
3,784
μ1 > 10 μ2 > 10
105
105
105
μ1 ∈ [0.1,1) μ2 ∈ [1,10)
100
50
10
5
1
0.5
-0.4
0
0.4
1

Figure S2. Significance of modified corrected Pearson correlation coefficients, (PCC′), as calculated by BigSur versus the Fisher formula, binned by gene expression. scRNAseq data were as described in Figure 3. Data points representing pairs of genes were divided into 21 bins based on the mean expression levels of each gene, and the results for each bin were plotted as in Fig. S1. The inset compares the PCC′-*p*-value relationship determined by BigSur (for genes with relatively high expression levels) with that predicted by the Fisher formula (dashed red line), showing that, for highly expressed genes, the two methods agree well.
