# Supplementary material for: Leveraging gene correlations in single cell transcriptomic data
Source: BMC Bioinformatics. 2024 Sep 18;25:305. doi: 10.1186/s12859-024-05926-z (PMC11411778; doi:10.1186/s12859-024-05926-z)
Supplement: Supplementary file 5 — Additional file 5: Figure S3. Distribution of equivalent PCCs. The p values obtained by BigSur for the melanoma cell line were transformed using the inverse of the Fisher formula to a set of “equivalent” PCCs. Since the Fisher formula operates on the absolute values of correlations, each calculated equivalent PCC was assigned the sign of the PCC′ value for the same gene pair. Equivalent PCCs provide a measure of correlation strength that can be compared across data sets with differing numbers of cells. They may be understood as a measure of how strongly correlated two normally distributed vectorswould need to be to produce the observed p value. Here, only those gene pairs judged significant by BigSur are shown. The fact that so many weakly correlated gene pairsare nevertheless statistically significant is a function of the long vector length in this experiment. [file 12859_2024_5926_MOESM5_ESM.pdf]

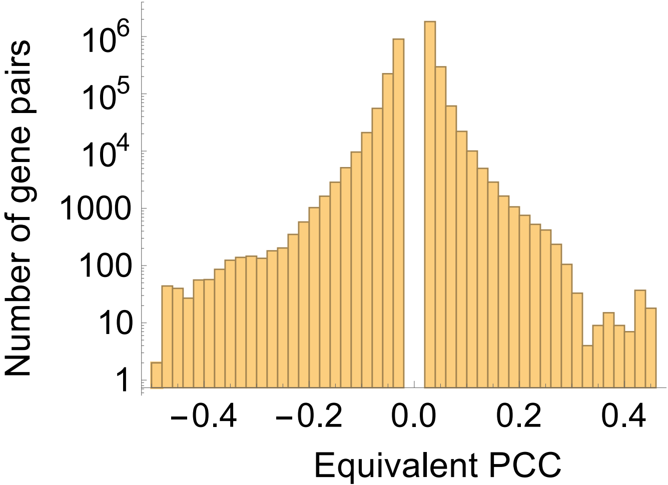
Number of gene pairs
10^6
10^5
10^4
1000
100
10
1
−0.4
−0.2
0.0
0.2
0.4
Equivalent PCC

Figure S3. Distribution of equivalent PCCs. The *p*-values obtained by BigSur for the melanoma cell line were transformed using the inverse of the Fisher formula to a set of "equivalent" PCCs. Since the Fisher formula operates on the absolute values of correlations, each calculated equivalent PCC was assigned the sign of the PCC′ value for the same gene pair. Equivalent PCCs provide a measure of correlation strength that can be compared across data sets with differing numbers of cells. They may be understood as a measure of how strongly correlated two normally distributed vectors (of any given length) would need to be to produce the observed p-value. Here, only those gene pairs judged significant by BigSur are shown. The fact that so many weakly correlated gene pairs (|equivalent PCC| <0.05) are nevertheless statistically significant is a function of the long vector length in this experiment (>8000 cells).
