# Supplementary material for: Leveraging gene correlations in single cell transcriptomic data
Source: BMC Bioinformatics. 2024 Sep 18;25:305. doi: 10.1186/s12859-024-05926-z (PMC11411778; doi:10.1186/s12859-024-05926-z)
Supplement: Supplementary file 6 — Additional file 6: Figure S4. Gene communities A and B from cell cluster 1.2. Green edges depict significant correlations. Transcription factor vertices are displayed as yellow boxes with gene names in blue. In the boxed insets the same graphs are overlayed in brown to highlight links supported by known protein-protein interactions. [file 12859_2024_5926_MOESM6_ESM.pdf]

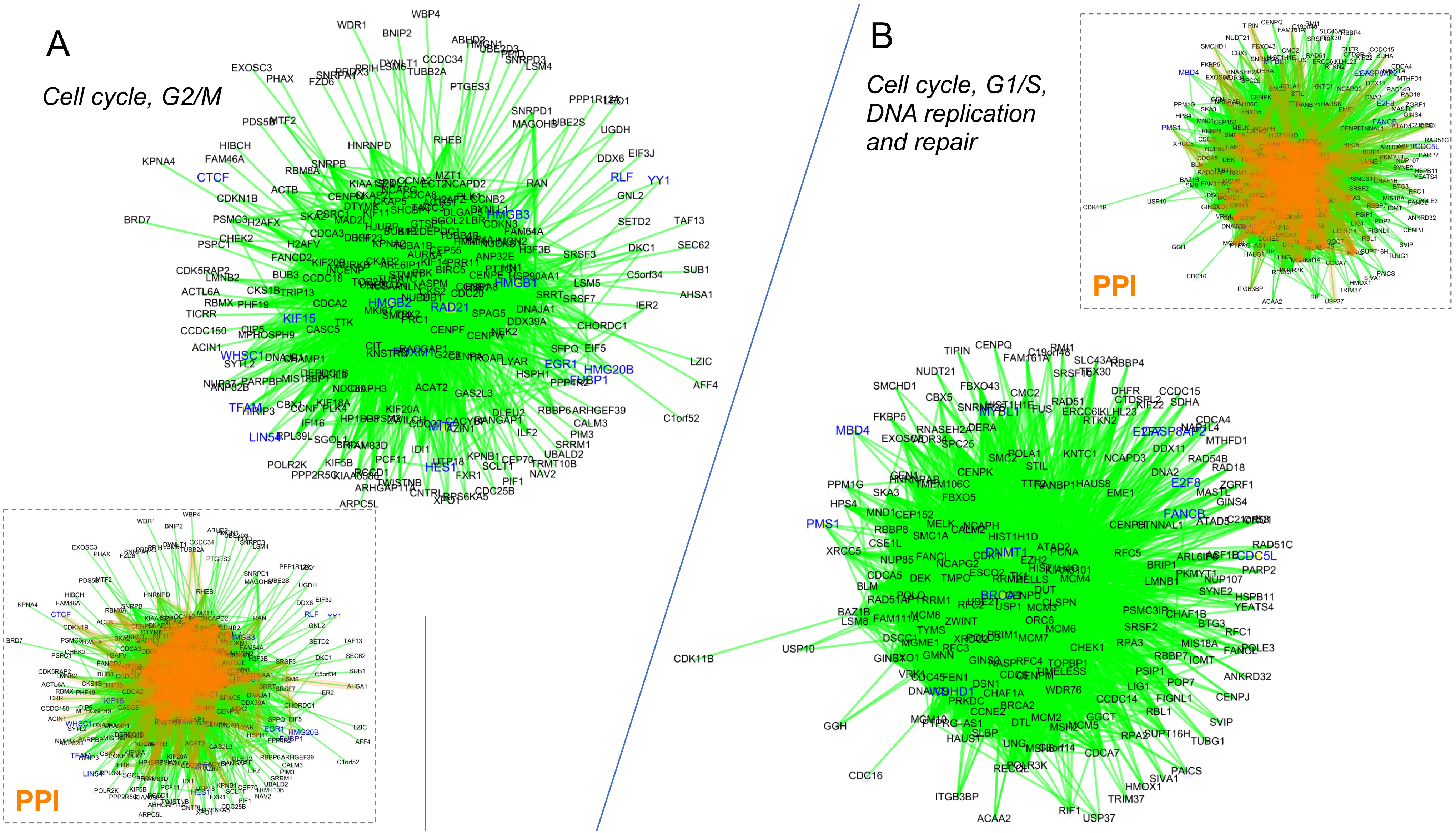
A
Cell cycle, G2/M
PPI
B
Cell cycle, G1/S, DNA replication and repair
PPI

Figure S4. Gene communities A and B (see Table 1) from cell cluster 1.2. Green edges depict significant correlations. Transcription factor vertices are displayed as yellow boxes with gene names in blue. In the boxed insets the same graphs are overlayed in brown to highlight links supported by known protein-protein interactions.
