# Supplementary material for: Leveraging gene correlations in single cell transcriptomic data
Source: BMC Bioinformatics. 2024 Sep 18;25:305. doi: 10.1186/s12859-024-05926-z (PMC11411778; doi:10.1186/s12859-024-05926-z)
Supplement: Supplementary file 7 — Additional file 7: Figure S5. Gene communities C, and D from cell cluster 1.2. Genes and links are highlighted as in Fig. S4. [file 12859_2024_5926_MOESM7_ESM.pdf]

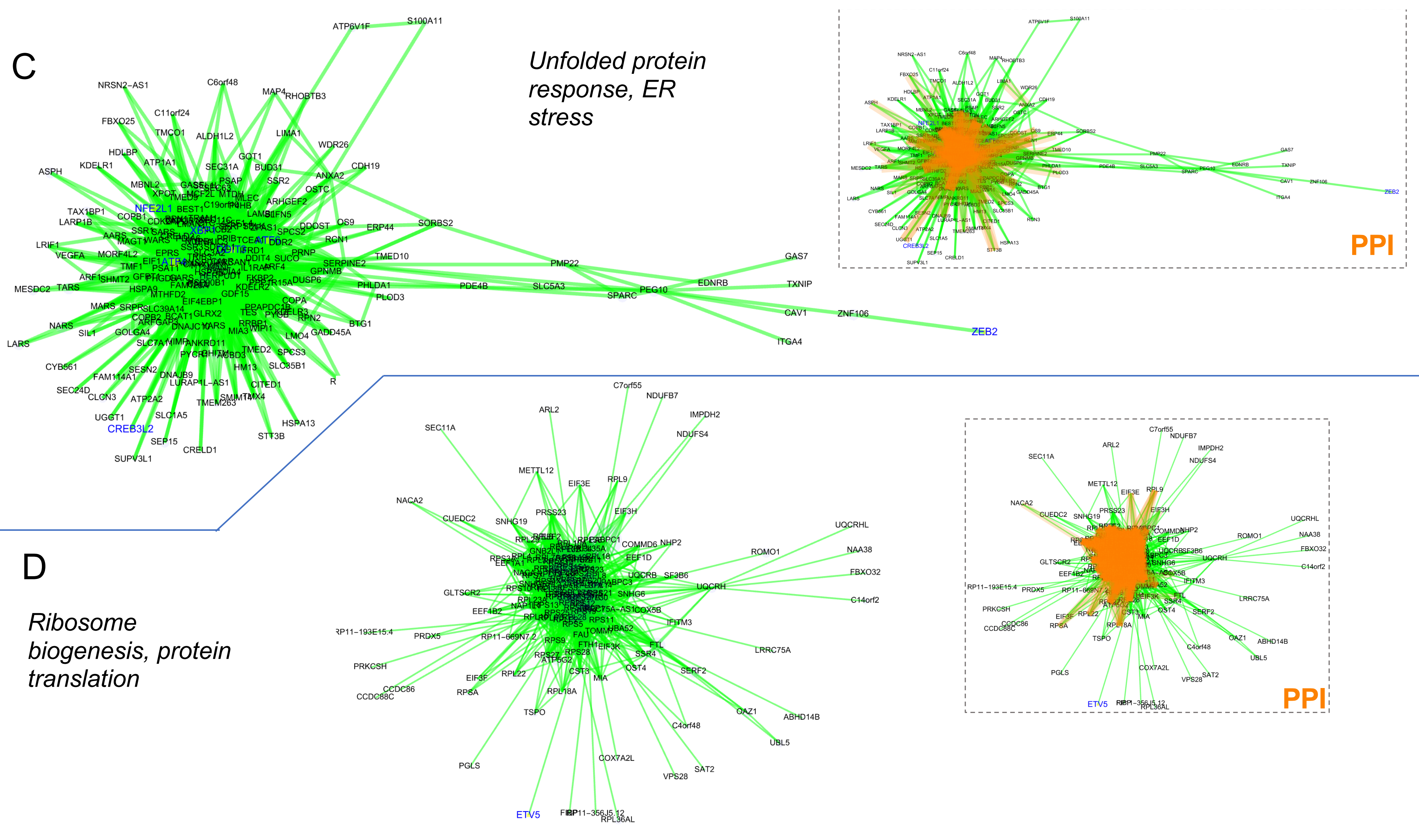

C
Unfolded protein response, ER stress
ATP6V1F
S100A11
NRSN2-AS1
C6orf48
MAP4
RHOBTB3
FBXO25
C11orf24
TMCO1
ALDH1L2
LIMA1
WDR26
HDLBP
GOT1
ASPH
KDELR1
ATP1A1
SEC31A
BUD31
CDH19
ANXA2
MBNL2
PSAP
SSR2
OSTC
ARHGEF2
NFE2L1
TAX1BP1
LARP1B
COPB1
OS9
DDOST
ERP44
SORBS2
XBP1
RCN1
LRIF1
VEGFA
MORF4L2
SUCO
PRNP
TMED10
SERPINE2
PMP22
GAS7
MESDC2
TARS
HSPA9
GPNMB
DUSP6
PHLDA1
PDE4B
SLC5A3
PEG10
EDNRB
TXNIP
SPARC
PLOD3
MTHFD2
GDF15
KDELR2
COPA
MARS
SLC39A14
GLRX2
TES
RPN2
CAV1
ZNF106
NARS
SIL1
GOLGA4
MIA3
WIPI1
LMO4
GADD45A
BTG1
ZEB2
LARS
SLC7A1
ANKRD11
TMED2
SPCS3
ITGA4
CYB561
SESN2
DNAJB9
HM13
SLC35B1
FAM114A1
SEC24D
LURAP1L-AS1
CITED1
CLCN3
ATP2A2
TMEM263
UGGT1
SLC1A5
HSPA13
CREB3L2
STT3B
SEP15
CRELD1
SUPV3L1
PPI
D
Ribosome biogenesis, protein translation
C7orf55
NDUFB7
ARL2
IMPDH2
NDUFS4
SEC11A
METTL12
EIF3E
RPL9
NACA2
EIF3H
CUEDC2
SNHG19
PRSS23
UQCRHL
COMMD6
NHP2
EEF1D
ROMO1
NAA38
UQCRB
SF3B6
FBXO32
UQCRH
C14orf2
GLTSCR2
SNHG6
EEF1B2
COX5B
IFITM3
RP11-193E15.4
PRDX5
RP11-669N7.2
RPS9
FTH1
EIF3K
FTL
LRRC75A
SSR4
PRKCSH
ATP5G2
OST4
SERF2
EIF3F
RPL22
CST3
MIA
CCDC86
CCDC88C
RPSA
RPL18A
OAZ1
ABHD14B
TSPO
C4orf48
UBL5
COX7A2L
PGLS
SAT2
VPS28
ETV5
RPL36AL
PPI

Figure S5. Gene communities C, and D (see Table 1) from cell cluster 1.2. Genes and links are highlighted as in Fig. S4
