# Supplementary material for: Leveraging gene correlations in single cell transcriptomic data
Source: BMC Bioinformatics. 2024 Sep 18;25:305. doi: 10.1186/s12859-024-05926-z (PMC11411778; doi:10.1186/s12859-024-05926-z)
Supplement: Supplementary file 8 — Additional file 8: Figure S6. Gene communities E, F, G and H from cell cluster 1.2. Genes and links are highlighted as in Fig. S4. [file 12859_2024_5926_MOESM8_ESM.pdf]

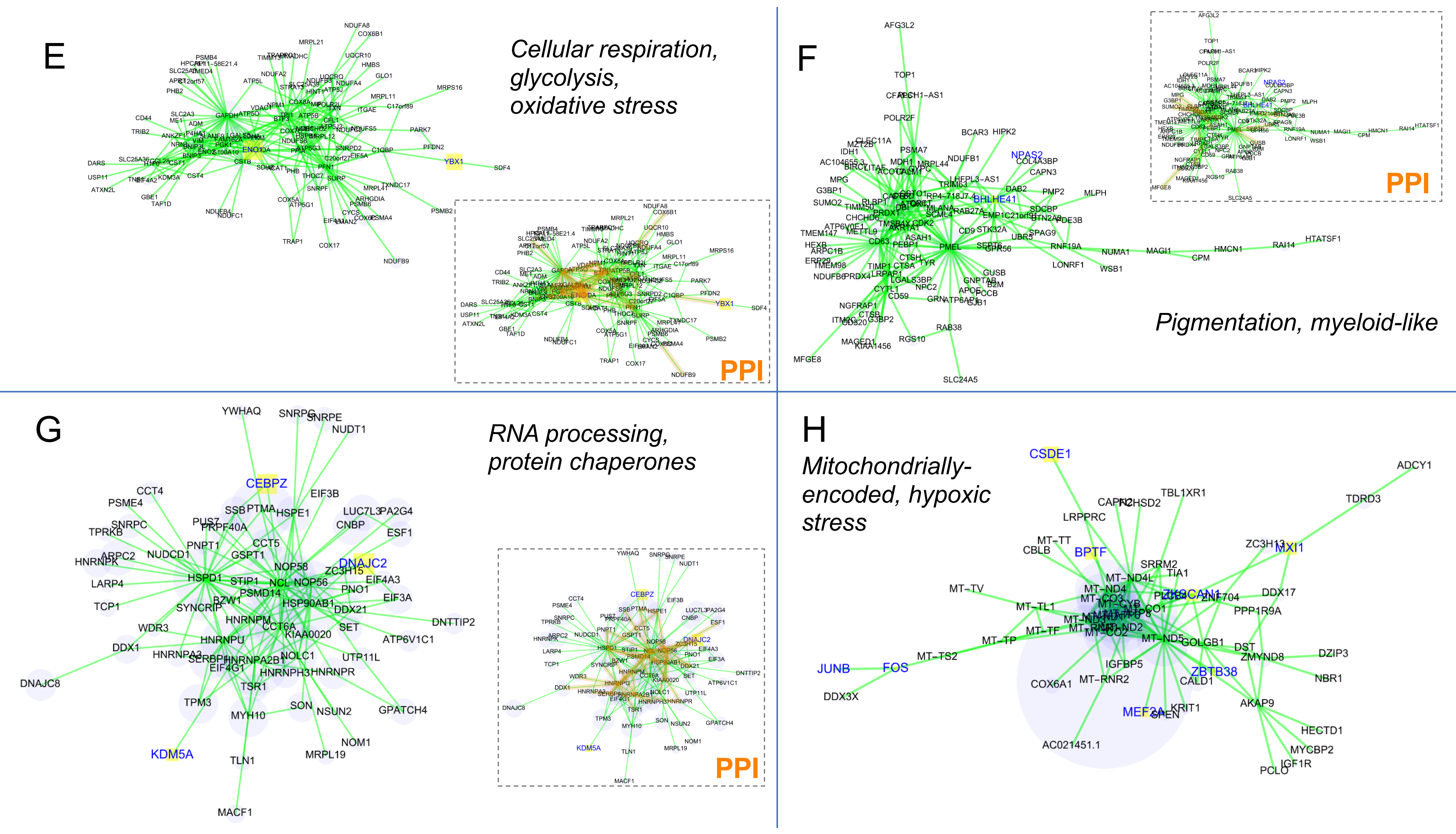

E
Cellular respiration, glycolysis, oxidative stress
GAPDH
ENO1
YBX1
SDF4
PPI
F
Pigmentation, myeloid-like
AFG3L2
TOP1
POLR2F
NPAS2
BHLHE41
PMEL
CD63
NUMA1
MAGI1
HMCN1
RAI14
HTATSF1
CPM
LONRF1
WSB1
MFGE8
SLC24A5
PPI
G
RNA processing, protein chaperones
YWHAQ
SNRPE
NUDT1
CEBPZ
EIF3B
CCT4
PSME4
HSPE1
LUC7L3
PA2G4
CNBP
ESF1
TPRKB
SNRPC
PRPF40A
PNPT1
CCT5
GSPT1
HNRNPK
ARPC2
NUDCD1
DNAJC2
NOP58
ZC3H15
LARP4
HSPD1
STIP1
NCL
NOP56
EIF4A3
PNO1
TCP1
PSMD14
BZW1
HSP90AB1
EIF3A
SYNCRIP
DDX21
DNTTIP2
HNRNPM
CCT6A
SET
WDR3
HNRNPU
KIAA0020
ATP6V1C1
DDX1
HNRNPA3
SERBP1
HNRNPA2B1
NOLC1
UTP11L
EIF4G1
HNRNPH3
HNRNPR
DNAJC8
TSR1
TPM3
SON
MYH10
NSUN2
GPATCH4
NOM1
KDM5A
MRPL19
TLN1
MACF1
PPI
H
Mitochondrially-encoded, hypoxic stress
CSDE1
ADCY1
TBL1XR1
CAPN2
HSD2
TDRD3
LRPPRC
MT-TT
CBLB
BPTF
ZC3H13
MXI1
SRRM2
TIA1
MT-ND4L
MT-TV
MT-ND4
ZKSCAN1
ZNF704
DDX17
MT-TL1
PPP1R9A
MT-TF
MT-TP
MT-ND5
GOLGB1
DST
MT-TS2
ZMYND8
DZIP3
JUNB
FOS
IGFBP5
ZBTB38
NBR1
DDX3X
COX6A1
MT-RNR2
CALD1
AKAP9
MEF2A
KRIT1
HECTD1
AC021451.1
MYCBP2
IGF1R
PCLO

Figure S6. Gene communities E, F, G and H (see Table 1) from cell cluster 1.2. Genes and links are highlighted as in Fig. S4.
