# Supplementary material for: Leveraging gene correlations in single cell transcriptomic data
Source: BMC Bioinformatics. 2024 Sep 18;25:305. doi: 10.1186/s12859-024-05926-z (PMC11411778; doi:10.1186/s12859-024-05926-z)
Supplement: Supplementary file 9 — Additional file 9: Figure S7. Gene communities I, K, L and M from cell cluster 1.2. Genes and links are highlighted as in Fig. S4. In communities L and M, links supported by known protein-protein interactions are highlighted in brown. [file 12859_2024_5926_MOESM9_ESM.pdf]

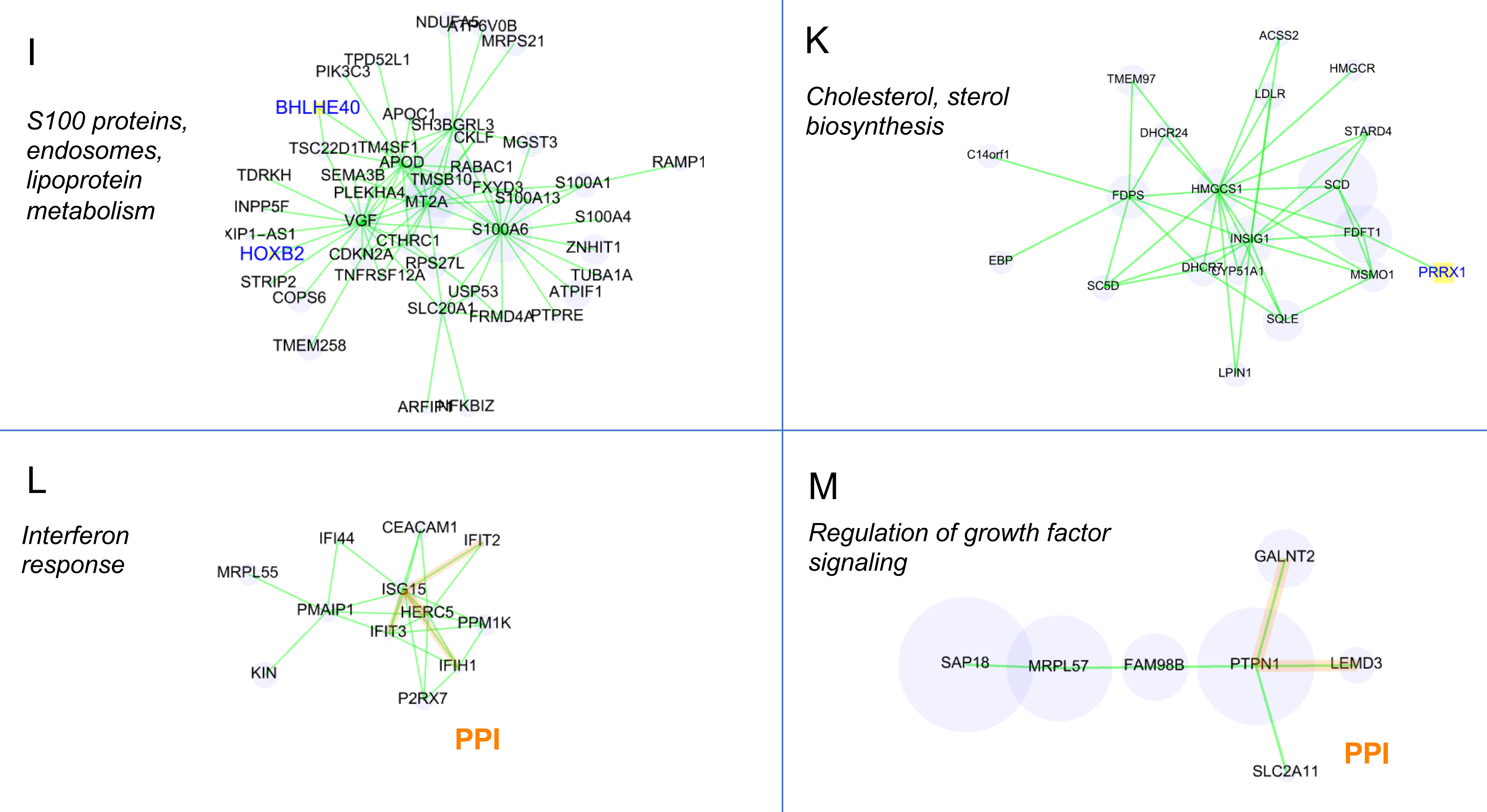

I
S100 proteins, endosomes, lipoprotein metabolism
NDUFA5
ATP6V0B
MRPS21
TPD52L1
PIK3C3
BHLHE40
APOC1
SH3BGRL3
CKLF
MGST3
TSC22D1
TM4SF1
APOD
RABAC1
RAMP1
TDRKH
SEMA3B
TMSB10
FXYD3
S100A1
PLEKHA4
MT2A
S100A13
INPP5F
VGF
S100A4
XIP1−AS1
S100A6
CTHRC1
ZNHIT1
HOXB2
CDKN2A
RPS27L
TUBA1A
STRIP2
TNFRSF12A
COPS6
USP53
ATPIF1
SLC20A1
FRMD4A
PTPRE
TMEM258
NFKBIZ
K
Cholesterol, sterol biosynthesis
ACSS2
HMGCR
TMEM97
LDLR
DHCR24
STARD4
C14orf1
FDPS
HMGCS1
SCD
INSIG1
FDFT1
EBP
DHCR7
CYP51A1
MSMO1
PRRX1
SC5D
SQLE
LPIN1
L
Interferon response
CEACAM1
IFI44
IFIT2
MRPL55
ISG15
PMAIP1
HERC5
PPM1K
IFIT3
IFIH1
KIN
P2RX7
PPI
M
Regulation of growth factor signaling
GALNT2
SAP18
MRPL57
FAM98B
PTPN1
LEMD3
SLC2A11
PPI

Figure S7. Gene communities I, K, L and M (see Table 1) from cell cluster 1.2. Genes and links are highlighted as in Fig. S4. In communities L and M, links supported by known protein-protein interactions are highlighted in brown.
