# Supplementary material for: Leveraging gene correlations in single cell transcriptomic data
Source: BMC Bioinformatics. 2024 Sep 18;25:305. doi: 10.1186/s12859-024-05926-z (PMC11411778; doi:10.1186/s12859-024-05926-z)
Supplement: Supplementary file 10 — Additional file 10: Figure S8. Graphical representation of positive correlations among genes significantly upregulated in lung epithelial cells treated with IL13 [76]. Of 419 upregulated genes, 313 form a single connected community in the treated cells, whereas 45 of those correlate in the untreated group. Green lines represent statistically significant positive correlations. Transcription factors are marked with blue text and a yellow box. [file 12859_2024_5926_MOESM10_ESM.pdf]

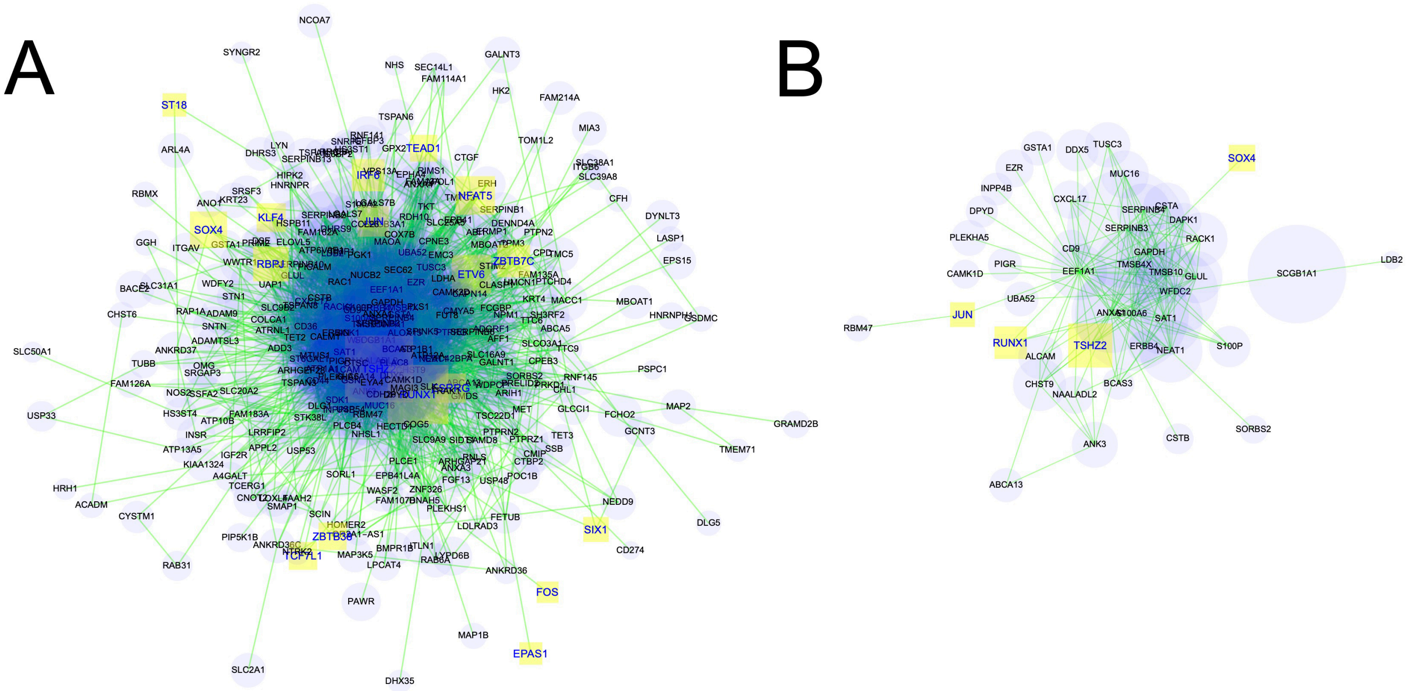
A
NCOA7
SYNGR2
NHS
SEC14L1
FAM114A1
GALNT3
ST18
HK2
FAM214A
TSPAN6
MIA3
ARL4A
LYN
DHRS3
TEAD1
TOM1L2
CTGF
SLC38A1
SLC39A8
RBMX
IRF6
NFAT5
CFH
DYNLT3
LASP1
EPS15
SOX4
KLF4
GGH
ITGAV
RBPJ
ZBTB7C
ETV6
BACE2
CHST6
MBOAT1
HNRNPH1
GSDMC
SLC50A1
PSPC1
USP33
MAP2
GRAMD2B
TMEM71
HRH1
ACADM
CYSTM1
PIP5K1B
RAB31
SIX1
CD274
DLG5
NEDD9
ZBTB38
TCF7L1
PAWR
MAP1B
FOS
EPAS1
SLC2A1
DHX35
B
GSTA1
DDX5
TUSC3
SOX4
EZR
MUC16
INPP4B
CXCL17
DPYD
SERPINB4
CSTA
DAPK1
PLEKHA5
SERPINB3
RACK1
CD9
GAPDH
PIGR
CAMK1D
EEF1A1
TMSB4X
TMSB10
GLUL
SCGB1A1
LDB2
UBA52
WFDC2
JUN
RBM47
ANXA1
S100A6
SAT1
RUNX1
TSHZ2
ERBB4
NEAT1
S100P
ALCAM
CHST9
NAALADL2
BCAS3
ANK3
CSTB
SORBS2
ABCA13

Figure S8. Graphical representation of positive correlations among genes significantly upregulated in lung epithelial cells treated with IL13 (Jackson et al., 2020). Of 419 upregulated genes, 313 form a single connected community in the treated cells (A) whereas 45 of those correlate in the untreated group (B). Green lines represent statistically significant positive correlations. Transcription factors are marked with blue text and a yellow box.
