# Supplementary material for: Leveraging gene correlations in single cell transcriptomic data
Source: BMC Bioinformatics. 2024 Sep 18;25:305. doi: 10.1186/s12859-024-05926-z (PMC11411778; doi:10.1186/s12859-024-05926-z)
Supplement: Supplementary file 11 — Additional file 11: Figure S9. Gene-gene correlation among genes that positively correlate with MUC16 in IL13-treated secretory lung epithelial cells. Genes were clustered using complete-linkage hierarchical clustering. Darker color indicates stronger correlation; white indicates no significant correlation [file 12859_2024_5926_MOESM11_ESM.pdf]

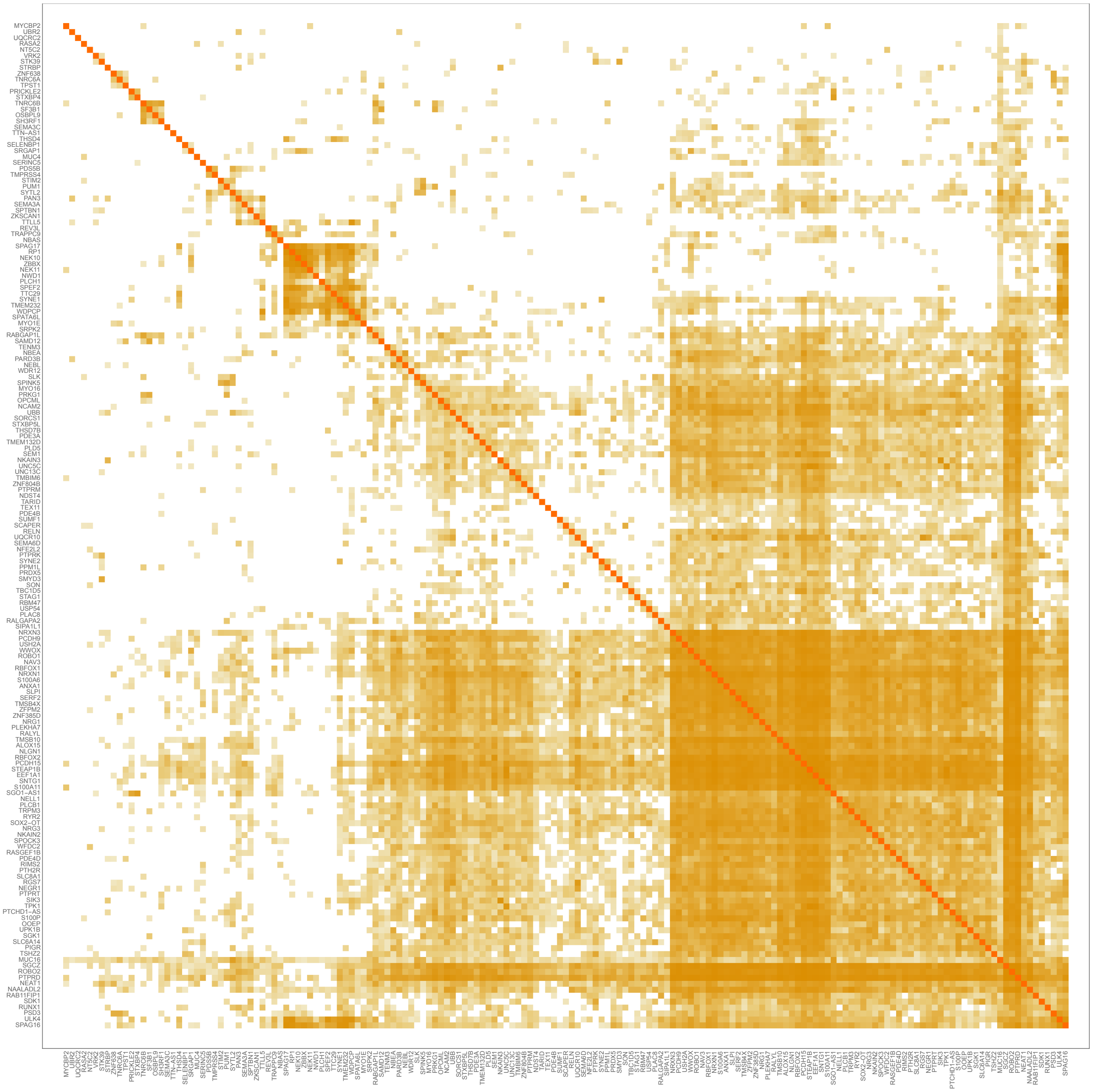

Figure S9. Gene-gene correlation among genes that positively correlate with MUC16 in IL13-treated secretory lung epithelial cells. Genes were clustered using complete-linkage hierarchical clustering. Darker color indicates stronger correlation; white indicates no significant correlation.
